# Interpersonal and Collective Affective Niche Construction: Empirical and Normative Perspectives on Social Media

**DOI:** 10.1007/s13164-022-00625-1

**Published:** 2022-03-18

**Authors:** Michiru Nagatsu, Mikko Salmela

**Affiliations:** 1 Center for Philosophy of Social Science (TINT), Practical Philosophy, Faculty of Social Sciences, Helsinki Institute of Sustainability Science, University of Helsinki, Helsinki, Finland; 2 Center for Subjectivity Research, Department of Communication, Faculty of Humanities, University of Copenhagen, Copenhagen, Denmark

**Keywords:** Extended mind, Collective affective niche construction, Extended emotion, Scaffolded affectivity, Social motivation, We-mode, Well-being, Social media

## Abstract

This paper contributes to the interdisciplinary theory of collective affective niche construction, which extends the extended mind (ExM) thesis from cognitive to affective phenomena. Although theoretically innovative, the theory lacks a detailed psychological account of how collective affectivity is scaffolded. It has also been criticized for its uncritical assumption of the subject qua the autonomous user of the affective scaffolding as disposable resources, abstracting away from embedded subjectivity in particular techno-political arrangements. We propose that the social motivation hypothesis, an account grounded in recent empirical and theoretical developments in psychology as well as in the classic theory of moral sentiments, will address the former criticism by explicating the basic mechanisms of human social orientation at work in collective affective niche construction. We also begin to address the latter normative criticism in mobilizing a so-called we-mode approach to collective emotion. To make these theoretical dialectics salient, we study social media as a case of collective affective niches, focusing on the impact on subjective well-being. Finally, we briefly identify promising future directions in building a normative theory of affective niche construction on the collective level.

## Introduction

Various aspects of the extended mind (ExM) thesis have been under extensive discussion in philosophical literature for more than two decades. A particularly interesting development is the extension of the ExM concept from the purely cognitive level to incorporate *affective* phenomena, such as moods, motivations, feelings and emotions (Carter et al. 2016). A promising line of enquiry is being pursued through *affective niche construction* theory (Colombetti and Krueger 2015), inspired by evolutionary biology and social psychology (Griffiths and Scarantino 2009; Sterelny 2010), as well as by the phenomenological tradition. In short, it is a theory that explains how an agent and her environment (including other agents) could be considered an integrated coupled system from the perspective of affective-functional analysis. The theory originates from the ideas of *niche construction* and *ecological inheritance* in evolutionary biology and ecology that criticized neo-Darwinianism for failing to appreciate the active role an organism plays in constructing its own environment thereby affecting its fitness and evolutionary trajectory (Lewontin 1983; Odling-Smee et al. 2013; see also Godfrey-Smith 2017). Affective niche construction theory utilizes these core ideas in theorizing the relation between human affective (as well as cognitive) psychology and cultural artefacts such as art forms (e.g., Maiese 2016; Hven 2019; Saarinen 2021). A particular aspect of affective niche construction theory has been criticized from a phenomenological perspective as naively presupposing the given subjectivity and autonomy of the agent using environmental resources to achieve her affective goals (Slaby 2016; Krueger and Osler 2019) indirectly address this normative critique in pointing out that some interpersonal niches, such as the Internet-enabled hyper-social technological kind, may make their inhabitants vulnerable and open to manipulation, which they call emotional *dysregulation*.^1^

Currently, there are two salient gaps in the literature. First, on the descriptive level, the *collective* dimension of affective niche construction is underdeveloped, relative to its individual counterpart. What are the distinct features and mechanisms of agent-agent compared to agent-object coupling? How do multiple agents collectively depend on each other in achieving affective experiences? Second, on the normative level there is no methodological framework within which to evaluate a given affective niche. The literature contains many goal-oriented terminologies such as function, regulation, management, engineering and design, but such terms cannot inform normative analysis because they are ambiguous with regard to who the relevant agents are, what their goals are and what their success criteria are. Both gaps need to be filled to inform a discussion on aspects such as whether and how the government should regulate the construction of these affective niches.

Our aim in this paper is to make these descriptive and normative gaps explicit, and to begin to fill them. More specifically, we have two goals. First, we will explicate the *collective* dimension of interpersonal affective niches, complementing Colombetti and Krueger’s (2015) analysis. Within the context of the *social motivation hypothesis* we will explain the tendency to construct collective affective niches in the first place, and to identify key psychological mechanisms underlying the process (Sect. 3). Our second goal is explicitly to discuss a normative dimension of affective niche construction, drawing on Slaby’s critique of the Colombetti and Krueger’ (2015) user-resource model (Sect. 4). We then adopt a so-called we-mode approach to collective intentionality in the case of collective affective niche construction, in order to introduce a necessary (but not sufficient) normative dimension. To flesh out these empirical and normative points, we broaden Slaby’s normative critique by examining a new case—the impact of social media on subjective well-being (Sect. 5), applying a social-motivation perspective in our interpretation of the reviewed studies. This enables us to build a mutually informative bridge linking highly theoretical and normative philosophical theories of extended emotion with empirical literature on the emerging techno-social niches, and thereby to understand their profound impact on our individual and collective life. We then address how we should reflectively evaluate the functions of social media as *collective* affective niches (Sect. 6). Section 7 concludes the article by summarizing our main argument.

## From extended minds to extended affectivity

Extended mind (ExM) approaches in the context of philosophy of mind date from the seminal work of Clark and Chalmers (1998), who suggested that some of our mental processes are physically realized in part by structures or processes located in one’s environment. Their famous example is Otto, a person with mild Alzheimer’s disease who uses his notebook for storing and retrieving important information, similarly to a blind person who uses a cane for orientation. Otto forms a coupled cognitive system together with his notebook (or in a modern version of the story, his smart phone), and in this does not differ functionally from a person who consults his or her memory for storing and retrieving information. This functional integration between Otto and his notebook allows them to be characterized as a single cognitive system. Clark and Chalmers focused on cognition, but they admitted that other mental states, including emotions, could also be extended.

Griffiths and Scarantino (2009) paved the way for the rise of extended emotion theories with their situated approach to emotion, which they formulate with reference to “transactional” accounts of emotion in recent social psychology. Griffiths and Scarantino summarise their view of emotion into four points, according to which emotions are:

1. Designed to function in a social context: an emotion is often an act of relationship reconfiguration brought about by delivering a social signal.
2. Forms of skillful engagement with the world which need not be mediated by conceptual thought.
3. Scaffolded by the environment, both synchronically in the unfolding of a particular emotional performance and diachronically in the acquisition of an emotional repertoire.
4. Dynamically coupled to an environment which both influences and is influenced by the unfolding of the emotion.

To elaborate, (1) people smile more often in the presence of others than when they are alone. This is an example of the role of social context in the production and management of emotions and the reciprocal influence of emotion on the evolving social context. (2) Instead of having a cognitive content that represents the emotion-eliciting situation, emotional content takes the form of an action-oriented representation in terms of what the environment affords to the emoter in the way of strategic engagement. Non-human animals (e.g. birds’ threat expressions) and prelinguistic children (e.g. embarrassment) have representations of this kind similarly to adult humans. (3) Scaffolding of emotions can be synchronic, diachronic, or both at the same time. For example, the wedding ceremony with rituals, music, and particular settings synchronically scaffolds the participants’ performances of their complementary affective roles. This “real-time socialisation” is supported by affective norms of wedding behaviour rooted in diachronic socialisation, in which participants internalise certain associations between occasions and emotions. (4) Finally, the dynamic coupling of emotions to the environment refers to the idea that emotional behaviours often have both expressive and negotiating functions. Anger in a marital quarrel is a case in point. Wilutzky (2015) has similarly argued that emotions in social contexts and their intentionality may be conceived of as pragmatic or epistemic actions that are aimed at achieving certain goals within a social context or at probing the social environment so as to extract or uncover important information.

Whereas highlighting similarities between situated emotion and extended cognition, Griffiths and Scarantino hesitate to characterise emotions as *extended*. This is because a situated perspective does not characterise the environment or the information it harbours as literally constituting a part of emotion. Extended Emotion theories proper, in contrast, directly apply the Extended Mind theory to emotions.

According to the theories of Extended Emotion, emotions extend beyond the brain and the body of the individual to objects of various kinds. These objects include: (a) material, artificial and technological tools (e.g., diaries, wedding rings, music, venues and sites, and scripts); (b) other individuals (e.g., caretakers, romantic partners, crowds and communities) and their emotion-expressive and embodied features (smiles, caresses, gestures, dancing) as well as symbolic or communicative interactions (e.g., conversations); and (c) complex structural, systemic or cultural tools, including ‘emotional climates’ or ‘emotional cultures’ (e.g., habits, conventions, feeling rules, rituals, socio-economic factors) (Krueger 2013, 2014; Slaby 2014; Colombetti and Roberts 2015; Krueger and Szanto 2016; León et al. 2019).^2^

Regardless of their details and the theoretical traditions upon which they draw, these accounts could be summarised as *externalist* with regard to emotions in recognizing functions of the environment that are indispensable if agents are to realize their certain affective states.

Our focus in this section is on Colombetti and Krueger’s (2015) account of extended emotion. These authors apply Kim Sterelny’s (2010) *epistemic* niche construction theory to emotions, which they describe as a promising direction. How do they justify the extended thesis along these lines?^3^ This theory is also interesting in that it highlights the distinction between *material* and *interpersonal* scaffolds of emotion more explicitly than any other theories of extended emotion, which nevertheless point to the same distinction. We suggest that this clarity sheds light on a fundamental problem in the entire extended-emotion approach insofar as these theories view material objects and other people on a par as scaffolds of individual emotions.

Niche construction theory proper belongs in the realm of evolutionary biology, which posits a more dynamic and reciprocal relationship between an organism and its environment than assumed in the standard theory. Accordingly, organisms actively modify the environment (including other organisms) and create a ‘niche’---natural selection pressures to which organisms are exposed, thereby changing their own evolutionary trajectories. Well-known examples include the beaver and the dam, and pine trees and forest fires. How exactly this theory differs from the standard theory of evolution, although debatable, is not important in this context in that our focus is, for the most part, on its application in the domain of culture. In particular, Sterelny (2010) explains how humans create the material and socio-cultural environment, which in turn enables, supports, enhances and moulds their cognitive capacities in profound and pervasive ways.

Sterelny’s (2010) account revolves around the analysis of three salient dimensions of the cognitive scaffolding: (i) *trust* in the resource; (ii) the *individualization* or *entrenchment* of the resource; and (iii) the *collectivity* of the resource. Trust, or trustworthiness, refers to the extent to which the agent has reliable access to and utilizes a certain environmental resource, either material or interpersonal, to realize her cognitive goals (e.g., publicly shared sources such as phrasebooks for communication and road signs for navigation). Individualization, or entrenchment, refers to the degree to which a resource has been adapted to an individual’s purposes and regular activities such that it has become *transparent* and incorporated into the acting and feeling self (e.g., a blind person’s cane for navigation). Collectivity refers to the interactions of individuals with collectively structured and realized environments that resist the discrete functional separation of the agent from her environment, including other agents.

A classic example of the collectivity of cognitive niches is how, in their spatial and temporal configurations of actors, Elizabethan theatres afforded an impressive theatrical repertoire: “A working actor might have to master 70 roles a year, perhaps 50 of which would be new, this while acting in six different plays a week.” (Sterelny 2010, p. 477) Historians (such as Evelyn Tribble) show that this demanding task (from an individual’s cognitive perspective) was enabled through scaffolding: “many aspects of the physical organization of the theatre itself, the thematic organization of the play, the minimal stage directions cueing a few but significant moments and the responses of the rest of the troop all work together to support the actor’s mastery of their roles.” (Sterelny 2010, ibid.) The key vision here is of a theatre troop as a *collective*, an *integrated system* of arrangements of space, sequences, scenarios and actors. Each actor is supported by the system, while also helping others to situate themselves within it. According to Sterelny, this functional interdependence among multiple agents and between them and their environment, rather than their ameliorative relations (“belonging to”), qualifies the case as an instance of collective cognitive niches.

Colombetti and Krueger’s (2015) account of affective niche construction applies Sterelny’s (2010) analysis to highlight the different ways in which scaffolding gives the agent the possibility of achieving certain *affective goals*, in contrast to her cognitive goals. They describe affective niches as “instances of organism-environment couplings that enable the realization of specific affective states” (p. 1160). However, they devote much of their analysis to the first two dimensions of collectivity (trust and individuation), neglecting the third somewhat. In the rest of this subsection, therefore, we complement their work, focusing on the collective dimension of interpersonal scaffolding.

Colombetti and Krueger (2015) note:

We spend time with partners, family, and friends because we enjoy their company and the pleasant feelings they bring about, and we engage in joint activities that are qualitatively enriched by the presence of others. (p. 1166)

It thus seems that other people are construed on a par with material scaffolding in the sense that they merely contribute to the agent’s individual affective goals. However, it is implicitly recognized that this type of collective affective niches is possible because of its *mutually beneficial* nature. Each individual benefits from the action or presence of other members of the group, while at the same time contributing to their positive affective states. There is a functional interdependence similar to what characterizes Sterelny’s collective cognitive niche. Hence, in the present context collectivity connotes a functionally interdependent and integral system.

Elsewhere, Colombetti and Krueger (2015, Sect. 5) briefly but more explicitly discuss the collective dimension of niche construction. Elaborating on “how items of material culture can scaffold the affective states not only of individuals but of social groups” (p. 1171), they attempt to find an affective analogy with Sterelny’s (2010) example of the Elizabethan theatres. Their examples concern the evolving practices of religious rituals in Christian churches: the priest having his back or face to the congregation (Roman Catholic practice); and the updated style of furniture and music (KingsGate Community Church in the UK), which may enhance “the sense of sharing and community of worshippers” or adapt to “the desires and preferences of a certain group of twenty-first century Christians” (ibid.). Collectivity in the latter passage only concerns the fact that these desires and preferences happen to be common among the members of the group, whereas in the former, something stronger (more collective) is implied in the expression “the sense of sharing”. However, how the sense of sharing experiences contributes to the positive affective states of individuals or the group is not further illuminated. One reason for this is that Colombetti and Krueger (2015) focus on the material aspects of the rituals as affective scaffolding, although it is assumed that other people play an important role in them. We therefore point to the need for a more explicit account of the collective dimension of interpersonal scaffolding beyond what Sterelny (2010) and Colombetti and Krueger (2015) offer.^4^ We propose such an account in the next section.

## The social motivation hypothesis

What are the functions that collective affective niches provide, and what are the mechanisms through which these functions are realised? We argue that social motivation serves as a useful theoretical framework within which to understand the human need and tendency to construct and inhabit collective affective practices and niches of various kinds. Furthermore, we suggest that it provides a bottom-up, empirically grounded explanation of how affective niches may sometimes harm participating individuals. Our account of social motivation in this section provides a basis for later discussion on the exploitative aspect of affective niches, which is the central concern of normative critiques (Sect. 4) and empirical studies (Sect. 5). Below we introduce our social-motivation hypothesis together with three affective mechanisms scaffolded by such motivation, drawing on Godman et al. (2014) and Salmela and Nagatsu (2017).

First, we argue that explicit analyses of the functions and mechanisms of *interpersonally* scaffolded affectivity are necessary not only because important affective (including material) scaffolding typically involves other agents or the anticipation of their reactions, but also because people tend to be drawn to interpersonal affective niches in ways that purely instrumental and strategic reasoning does not explain. Our main idea is that there is a psychological disposition we characterise as ***social motivation***, the role of which is to orient individuals towards affiliative stimuli that yield social rewards (affect) and enable the formation of social bonds (Godman et al. 2014).^5^ The social motivation hypothesis is succinctly summarised by Coralie Chevallier and colleagues, who defend it in a different context.

Social motivation can be described as a set of psychological dispositions and biological mechanisms biasing the individual to preferentially *orient* to the social world (**social orienting**), to *seek and take pleasure* in social interactions (**social reward**), and to work to *foster and maintain* social bonds (**social maintaining**). (Chevallier et al., 2012, p. 231).

The idea is that affiliative stimuli initially become “incentively salienced” due to people’s biased capacity for experiencing such rewards. The initial link between affiliative stimuli and social reward is then strengthened by means of associative learning. In other words, because rewards are typically attached to certain affiliative stimuli, they become increasingly associated such that they yield (social) motivation in expectation of distinctively social rewards. The intrinsic rewards afforded by affiliation and sociality orient people become ever more strongly towards the social world. Nevertheless, the kind of affiliative stimuli people seek and find rewarding depends crucially on their past experiences and learned associations. We therefore do not claim that all social stimuli are rewarding and sought-for. We also point out that social motivation is a *bias* that enhances the fitness of humans as a species, and may have negative welfare consequences for individuals, as we will show in Sect. 5.

We suggest that social motivation underlies three types of affective mechanism that operate in social interaction, rendering it rewarding for its own sake: affective synchrony, collective effervescence, and fellow-feeling. These mechanisms are closely related and mutually reinforcing.

### Mechanism #1: affective synchrony

An association between synchronised behaviour and positive interpersonal affect has been established in several studies. These affective rewards, which include feelings of solidarity, rapport, affiliation and interpersonal liking, as well as prosocial and cooperative motivation, are associated with different types of synchrony among the interacting agents. Examples include synchronised motor representations (Rizzolatti and Craighero 2004), body postures and gaze patterns (Shockley et al. 2009), speech patterns (Fowler et al. 2008), facial expressions (Chartrand and Bargh 1999) and heart rate (Vikhoff et al. 2013). Moreover, synchrony yields affective rewards even when it is accidental rather than purposeful, such as when individuals walk, tap their fingers, rock chairs, and so on, or perceive figures or sounds (e.g., Hove and Risen 2009; Miles et al. 2009). There is little opportunity for feedback in these cases, and little history of social interaction, nor is there any reason to suppose the presence of shared intentionality. Nevertheless, individuals who engaged in synchronous behaviour report stronger feelings of interpersonal connectedness, rapport and affiliation, and they sometimes cooperated more when met with social dilemmas than people in asynchronous conditions (Wiltermuth and Heath 2009). The implication here is that behavioural synchrony constitutes a basic building block for daily interactions with others, and the inherently rewarding aspect of such behaviour explains why people are *prima facie* motivated to participate in joint actions that involve aspects of synchrony (Godman 2013; Godman et al. 2014).

While existing research confirms that mimicry in its various forms (facial, emotional, behavioural) increases mutual liking, rapport, empathy, and prosocial motivation whenever it occurs, there is growing evidence that mimicry is heavily influenced by top-down processes rather than a simple perception driven process. Thus, recent theories of facial and emotional mimicry suggest that it substantially depends on higher-level processes such as goals, appraisals, and meaning-making (Fischer and Hess 2017). “We do not mimic facial features per se, but the meaning of these movements, which are related to an emotional or social signal. Emotional mimicry thus is goal driven, rather than stimulus driven.” (Ibid., 151). Similarly, behavioural mimicry is not merely a consequence of the link between perception and behaviour, but rather a non-consciously activated behaviour that is motivated by affiliation goals and sensitive to the intention of a given behaviour (facial expression or gesture) as well as the context in which it occurs (Arnold and Winkielman 2020; Duffy and Chartrand 2015). All these studies highlight the importance of social context and previous interpersonal connections to mimicry.

The fact that synchronous behaviour is a regular feature of joint action, as Pacherie (2014) and Tollefsen (2014) observed, implies that this mechanism of shared affect operates in most such instances. Of course, the affective rewards of synchrony may not suffice to render the overall hedonic quality of a joint action positive, as when two or more people carry a heavy sofa, for example. Nevertheless, if individuals experience even contingent behavioural synchrony as rewarding in its own right, they could be expected to engage purposefully in forms of social interaction in which synchrony and the adjacent rewards are produced. This seems to happen in ritualistic contexts such as play, games, singing, dancing, worship and other ceremonies.

### Mechanism #2: collective effervescence

The emergence of shared emotions in ritualistic social interaction has been analysed within the Durkheimian tradition of sociology. These theorists suggest that the function of all social interaction, whether people are aware of it or not, is to produce affective rewards for the participants and to reinforce social cohesion in the group (e.g. Durkheim 2001[1912]; Collins 2004, 2014; Summers-Effler 2007). This ultimately serves to increase the adaptiveness of the community (Collins 2004). On the proximate level, people seek an affective boost from interactions they have experienced as rewarding and avoid emotionally draining interactions. Indeed, according to ritual theory, the fundamental drive to maximise our emotional energy is “behind individual behaviour, group activity, culture, and networks” (Summers-Effler 2007, p. 139).

Emilé Durkheim is the *locus classicus* of the ritualistic tradition. His analysis of the emergence of collective effervescence in the gatherings of people with common beliefs and convictions was the foundation from which later sociological accounts emerged, including Randall Collins’s theory of the emergence and reproduction of collective emotions in interaction rituals. The prime ingredients of a ritual include a specific group of people who are assembled in the same place, separated from those who are excluded from it. Second, there is shared intentionality in the form of the participants’ shared attention to the same object or event and their mutual awareness of this common focus of attention. Finally, the participants share a common mood or emotion. These initiating affects spread and intensify in the group’s interaction rituals, such as chants, songs, dances, or games, by means of emotional contagion and the rhythmic synchronisation of bodily responses, as well as through the group members’ reflective awareness of their shared experience. Collins refers to the result of this as collective effervescence, in other words heightened intersubjectivity in which “[t]he key process is participants’ mutual entrainment of emotion and attention, producing a shared emotion/cognitive experience” (Collins 2004, p. 48). A successful interaction ritual produces emotional energy (confidence, enthusiasm and positive self-feelings) among the participants, collective symbols (emblems, signs, slogans, buzzwords, ideas and other representations) infused with emotional energy, feelings of solidarity, and standards of morality exemplified in respect for the group and its symbols and anger at violations against them.

From the perspective of this article, the main contribution of ritualistic theories is their detailed descriptions of how the emotionality of ritualistic activities builds on the elements of engagement in joint activity, such as the separation of the participants from the others, their shared focus of attention, and an initial common mood or emotion. The emergence of collective effervescence is a dynamic process emanating from the mutually reinforcing interplay of ritual ingredients. First, affective rewards from behavioural synchrony and alignment may constitute one source of the initial shared affect that spreads and intensifies in the course of an interaction ritual. Another source is the internal goals and standards of excellence expected in rituals, such as maintaining the same rhythm in communal dancing or singing, doing a difficult part in a joint performance and completing the performance rather than interrupting it. Shared emotions emerge as rational responses to jointly succeeding or failing to comply with the internal norms of rituals. (Parkinson et al. 2005; Helm 2008; Salmela 2012).

This motivation to engage in joint actions in rituals and the subsequent rewards are the key to unpacking what theorists mean by “emotional energy” that motivates people to return to those activities, either with the same interactors or with other masters of the same activities. However, this still does not explain why successful rituals create feelings of solidarity and interpersonal liking. What is it in sharing emotions with others that has these effects? We argue that Adam Smith’s classic theory of fellow feelings addresses this question.

### Mechanism #3: fellow-feelings and the correspondence of sentiments

According to Adam Smith (2002[1759]), the prospect of a reward produced in a two-stage psychological mechanism explain prosociality and the tendency to do things together with others rather than alone. Fellow feeling is a form of empathy that allows one to feel, to a lesser degree, what another person feels through the imaginative placing of oneself into his or her situation. This active consciousness of another person’s affective state produces corresponding sentiments between the empathising and the empathised subject. Becoming aware of this correspondence of sentiments gives these subjects an additional source of pleasure. Moreover, such awareness is rewarding regardless of whether the given sentiment is pleasurable or painful, and that the pleasure from the correspondence may outweigh any painful sentiment such that people seek such correspondence whenever possible. In Smith’s words, the awareness of correspondence in sentiments “enlivens joy and alleviates grief.” (Smith 2002, p. 18).

Sugden (2002) claims that Smith is best interpreted as arguing for a general hypothesis that any correspondence of sentiments, whether caused by fellow feeling or some other mechanism such as sharing emotions grounded on a shared concern, is a source of pleasure, and the lack thereof is a source of pain. In a nutshell, the Smith-Sugden hypothesis states that this reward from the correspondence of sentiments is primarily responsible for the tendency in individuals to *align their sentiments with those of others*. Accordingly, the feelings of solidarity and interpersonal liking that ritual theorists highlight are subjective manifestations of this specific reward system.

Thus, the Smith-Sugden hypothesis explains phenomena identified by theorists of ritual and social interaction, without specifying their psychological mechanisms involved. As Collins argues, the initial common affects of individuals may be positive or negative, but the resulting collective emotional experience always has a positive quality or side to it. For instance, the ritual of funerals allows bereaved family members and friends of the deceased to express their grief to each other and to be consoled by the shared experience. The same mechanism operates in positive emotions such that awareness of the shared experience amplifies its positive quality. As people know from experience, watching a game involving their favourite team is more exciting and fun when done together with fellow fans, either at the stadium or at home via TV, than watching it alone. The pleasure in “feeling the same” emerges when individuals become aware of their shared sentiments, and the anticipation of those pleasant feelings reinforces future interactions in which such sentiments are likely to be experienced.

## A critique of interpersonal affective niche construction

Having supplemented an empirical psychological account of interpersonal affective niche construction, we now present a two-part normative critique of the models of interpersonal affective niche construction produced by Colombetti and Krueger (2015), and Krueger and Osler (2019). First, we introduce Jan Slaby’s critique of accounts related to extended emotions, and that of Colombetti and Krueger (2015) in particular. Slaby argues that these theories subscribe to a “user/resource model” that misconstrues the individual as an autonomous “user” of the external environment as his or her affective resource, neglecting the complex ways in which socio-technologically engineered environments invade the mind. We observe that Krueger and Osler (2019) address Slaby’s criticism by emphasising the reciprocal influence of individuals who both act on and are acted upon by others in interpersonal affective niches. However, we suggest that their notions of “we-experience” and “we-space” are still ambiguous with respect to the participants’ autonomy and well-being -- concerns Slaby raised. We, therefore, introduce a distinction between *collective* and *interpersonal* affective niches, which we draw from the literature on collective intentionality, in particular from Raimo Tuomela’s distinction between *I-mode* and *we-mode* collectivity. In short, we suggest that collective affective niches operating in the we-mode respect the autonomy of the participants as a group and also provide them with more social rewards. We believe that our we-mode approach offers a more robust criterion for the political evaluation of interpersonal affective niches than Slaby’s political philosophy of mind.

### Slaby’s critique of the Extended Mind approach

Jan Slaby’s article “Mind Invasion: Situated Affectivity and the Corporate Life Hack” (2016) makes explicit an underlying assumption, which he refers to as a “user/resource model”, in the Extended Emotions approach and in that of Colombetti and Krueger (2015) in particular. He argues that this approach is misguided in taking the individual as an autonomous ‘user’ of the external environment as his or her affective ‘resource’, without questioning how individual affective states are enabled and constrained by environmental structures, practises, norms, technologies and institutions. Thus, he argues that socially scaffolded affectivity is “prior to and formative of individual emotion repertoires and affective-bodily styles, which themselves are part and parcel of shifting structures of subjectivity.” (Slaby 2016, 2).

As an exemplary case, Slaby analyses the ways in which the contemporary corporate workplace with its affective practises and norms “invades” and “hacks” the minds of its employees by involving them in draining and exploitative affective-bodily styles that require them to be alert and attentive to social stimuli such as emails 24/7.

They establish affective habits of rampant attentiveness, lead to hectic efforts in staying tuned, kindle anxieties of disconnection, and often provide sustained access to a sphere of activity and relatedness that quickly exceeds what an individual employee can reliably process (p. 11).

The term “mind invasion” is intended to capture the sense that it is not entirely up to individuals to engage in such affective practises and styles even if they have the impression that they are making such a decision in pursuit of their own overall interests.

In a recent paper, Krueger and Osler (2019; in particular pp. 225–226) describe some aspects of mind invasion in their careful analysis of emotional *dysregulation* by Internet-enabled techno-social affective niches. They observe that, as much as people trust and individualise these affective niches, crucial elements of that environment are beyond their agential control, making them affectively vulnerable and open to manipulation. However, the authors do not offer a clear solution to the problem of emotional dysregulation. Slaby (2016), on the other hand, offers an explicitly normative proposal, which he calls a *political philosophy of mind*: it means becoming aware of the complex socio-normative patterns that enable and constrain individual mental states in particular contexts, and submitting these patterns to critical scrutiny. This allows the use of *reflective endorsement* to distinguish exploitative from empowering and enriching affective niches.

We find Slaby’s critique of the user/resource model and his analysis of the socially and technologically embedded nature of human affectivity insightful, which we interpret as a variation of the normative work on the politics of embodied subjectivity (e.g., Ahmed 2014). Even so, we believe that his focus on the corporate world provides a one-sided picture of the issue. True enough, negative phenomena such as rampant attentiveness to social stimuli and anxieties of disconnection from others may emerge in all social groups, not merely at corporate workplaces. However, they seem less prevalent and salient in contexts such as community-based volunteer groups, social clubs, hobby groups, associations and so on, the purpose of which is not to make profit but to facilitate joint activities that the participants find valuable for non-pecuniary reasons. Many of these activities, such as singing, dancing, playing and doing sports or communal work together are rewarding for their own sake. They paradigmatically occur face-to-face in designated locations and at scheduled times, although they are increasingly coordinated and implemented on the Internet-enabled platforms (Krueger and Osler 2019): this is particularly prevalent during the current Covid-19 pandemic. In addition, membership of these social groups is based on voluntary commitment rather than predominantly economic incentives that motivate people to stay at an exploitative workplace if better alternatives are not available.

Closer consideration of the variety of social groups indicates that the drive to interact with others is a fundamental human motivation, as we have argued in Sect. 3 above. It is a motivation that is not completely amenable to control or rational calculus, even if designers of platforms and environments of social interaction (both offline and online) seek to exploit and harness it for their own purposes. However, volunteer organisations and social groups provide examples of social environments in which participants are simultaneously “users” and part of the communal “resource” in *reciprocal* and *cooperative* collectives with shared goals. In other words, these individuals are functionally interdependent. Notably, the participants of these groups contribute to the construction and maintenance of their affective niches, instead of being mere recruits to niches largely designed by others, analogous to the actors in Elizabethan theatres in Sterelny’s (2010) example. This allows them to recalibrate the niches if they are dissatisfied with them.

### A we-mode approach to collective affective niches

We therefore suggest that a major problem in models of interpersonal affective scaffolding and niche construction concerns their unanalysed nature of collectivity. The basic idea behind affective scaffolding is that some affective experiences, such as shared emotions, are possible only through participation in social interaction. In such cases, “we can say that an individual is able to realize a type of affective experience that they could not have had alone; the other person is part and parcel of that experience.” (Krueger and Osler 2019, p. 218).

Such ‘we-experiences’ take place in what these authors call a *we-space*, “a kind of affective niche: a felt sense of shared space which opens new possibilities, actions, interpersonal understanding, feelings, and connection for those involved” (ibid., p. 219). Nevertheless, questions concerning (i) the kind of shared experiences that are realized in these spaces and (ii) what it is that affords these experiences remain open (cf. Krueger 2011).

A major distinction concerning types of collectivity in contemporary literature on the philosophy of collective intentionality is that between *I-mode* and *we-mode* collectivity (Tuomela 2007, 2013). Tuomela argues that individuals may function in a group context in either the I-mode or the we-mode. When they act or express attitudes in the I-mode they function as group members, but their commitment to the relevant action or attitudes is private, in other words it is based on their goals as private persons: for instance, two individuals have the same destination and therefore they share the same taxi instead of each taking their own. The I-mode is the weakest form of collective intentionality, “and it simply requires that the agents have an attitude with the same content and mutually believe that they have it” (Tuomela 2013, p. 6). The we-mode is stronger. It requires that individuals intend to act together or have attitudes as a group for the same authoritative group reason, and that they conceive of themselves as group members who are bound by and committed to what is collectively accepted and subject to collective commitment in the group. An example of a we-mode group is a team whose individual members have collectively committed to the same goal, which they pursue together for the same group reason grounded in their shared goal. Between the plain I-mode and the full-blown we-mode, there is a type of collective intentionality that Tuomela refers to as either the *pro-group I-mode* or the *weak we-mode*. It resembles the full-blown we-mode in the sense that individuals in this mode of collective intentionality have shared goals, such as going to see a movie together, which they pursue together as a group. Nevertheless, their reasons for participating in the shared activity are still private and therefore possibly divergent, based on personal rather than collective commitment, and thereby resembling plain I-mode collectivity.

In what follows we mainly refer to Tuomela’s framework, but the points we make are more general. For example, consider another prominent distinction in the literature, that between team-reasoning and individual-reasoning, which draws on game theory rather than the philosophy of action (Bacharach 2006; Sugden 1993; Gold and Sugden 2007). According to this account, even a weak we-mode collective, which Sugden calls a community of advantages, tends to develop certain shared normative standards of conduct. Bruni and Sugden (2013) characterize these as virtues, which is not very different from what Tuomela calls group ethos in we-mode collectives.

Considered in light of the distinction between I-mode and we-mode collectivity, constructs such as interpersonal affective scaffolding and niche construction seem to have features of the I-mode. As Colombetti and Krueger (2015) state, “the interpersonal domain is a realm that we actively manipulate to alter our affective states” (p. 1166). This looks like a clear case of I-mode activity in which the social environment is perceived from the perspective of individual emotion regulation. Individuals may even have the same goal in manipulating their social environments: to experience pleasant feelings qualitatively enriched by the presence of others, as Colombetti and Krueger observe. Even so, insofar as these goals merely overlap private goals, they only manifest plain I-mode or pro-group I-mode collectivity rather than full-blown we-mode collectivity. True enough, Krueger and Osler (2019) characterize the kind of we-experiences that individuals engaging in we-spaces may have as being “in the first-person plural, as an experience that ‘we’ have” (p. 218). As phenomenologists, they take this kind of experience as a criterion of a robust ‘we’. However, it seems to us that these ‘we-experiences’ sometimes come cheap and easy, without involving strong forms of collectivity. This is the case for instance in León et al.’s (2017) example of the passer-bys’ shared joy about the skillful performance of a street musician: “One might share with another passer-by the delight of witnessing a violinist playing on the street, and a sharing look and a smile might make it transparent that the delight is shared.” (p. 14). We think that a notion that is not capable of distinguishing cheap and light forms of “we-experience” from more robust ones is not very fruitful theoretically. Some of the social-motivation mechanisms we discuss in Sect. 3, such as fellow-feeling, are at work here, but this does not mean that we-mode collectivity is involved.

We argue that it is worth distinguishing the types of collectivity involved in we-experiences for two reasons: first, we-mode collectivity leads to more robust and reliable social rewards from social interaction than I-mode collectivity (see Sect. 3); and second, we-mode collectivity addresses the normative worry concerning manipulation and exploitation by socio-technological niches.

Beginning from the first point, the mechanisms of social reward -- affective synchrony, collective effervescence, and pleasure from correspondence in sentiments -- clearly operate in some way in all social interactions, whatever the degree of collectivity. Shared goals are not required for the rhythmic synchronization of behaviors, nor for experiencing collective effervescence with others as individuals may have different reasons for participating in rituals, and fellow-feeling allows the empathic sharing of another person’s emotion and the experience of rewarding correspondence in sentiments without sharing goals. Indeed, feelings of togetherness emerging on social-media platforms generally seem to be cases in which emotional convergence emerges through empathy, and several platforms appear to be well-suited to this type of affective sharing (see Osler 2020). However, the use of these platforms are not explicitly aimed at joint action in the pursuit of shared goals, although they can facilitate --and indeed have recently been recruited to serve -- such goals^6^.

Despite these reservations about Internet-enabled environments as platforms for emotional sharing, there is a type of social interaction in social media in which we-mode collectivity is common, namely discussion groups in which participants post, discuss and share news and stories of their mutual interest. Discussion is a form of joint activity even if it is not typically conducted with the express aim of achieving a particular goal or outcome, such as reaching a rational consensus on the topic of discussion as in the classic Socratic dialogues, or a decision on joint actions to be pursued together as in social and political movements. Most tellingly, participants of discussion groups could be individuals who belong to community-based volunteer groups, social clubs, hobby groups, religious communities, ideological associations and the like, the purposes of which, apart from discussion, may include the facilitation and coordination of joint activities, both offline and (especially during the current pandemic) online. These activities may be based on the participants’ collective commitment to a shared goal, value or ideal -- a *group ethos* that normatively guides and constrains the actions and responses of group members on the platform. By virtue of we-mode collectivity, emotional sharing among the participants takes the form of a projectible pattern of rationality in their responsiveness to the world, as Helm (2008) points out. Thus, they experience shared joy when the group ethos is advanced, shared fear when the group ethos is threatened, shared anger at those perceived to be responsible for the threat to the group ethos, and so on (Helm 2008; Salmela 2012). It is worth pointing out that shared emotions of this kind are not merely contingent responses to events relating to the shared ethos, they are rational responses that both express and partially constitute the group membership. Given that they are possible only in collective affective niches with we-mode collectivity, we argue that shared emotions of this kind may provide more robust and reliable social rewards than affective niches with weaker types of collectivity.

Second, we argue that we-mode collectivity is also better equipped to resist the manipulation and exploitation of the less powerful by the more powerful, which Slaby’s normative critique of affective niche construction problematizes.^7^ The major difference between collective affective niches with we-mode as opposed to I-mode collectivity concerns the reasons why individuals participate in these groups. In the former their reasons are based on a collective commitment to a shared group ethos, whereas in the latter they are divergent private reasons of the participating individuals. Collective commitment is the normative glue that binds the members of a we-mode group together in terms of ethos, and aligns group-based rights and member obligations. No such normative glue or structure is readily available in I-mode groups, although these groups may develop features of we-mode structure in the course of time. There can also be hybrid groups with both we-mode and I-mode participants, although we can treat such cases as we-mode groups if there is a common understanding among the group members of a we-mode structure within the group.^8^

This is of significance in that the we-mode structure allows the occupants of a collective affective niche to contribute to its construction and maintenance -- within the limits of parameters set by commercial service providers such as Facebook in the case of online groups -- in a manner that is not feasible in interpersonal affective niches that only serve individual purposes. Whereas the former co-develop and share the group ethos, patterned on norms of *reciprocity* and *cooperation* in shaping their niche, occupants of merely interpersonal affective niches are not strongly governed by these group-oriented norms. This lack of (or weaker) collective norms in the latter type of niches may, in turn, result in the manipulation or exploitation of individuals with fewer resources or less bargaining power, which would be counteracted by norms of reciprocity and cooperation in collective niches. Other things being equal, these considerations render collective affective niches normatively preferable to their merely interpersonal equivalents. From this perspective, we would construe Slaby’s critique of “corporate life hack” as a call for more robust collectivity in the workplace.

## Affective niches and subjective well-being: the case of social media

Now that we have provided a positive account of affective niche construction based on the social motivation hypothesis (Sect. 3), and a normative account of the collective (as opposed to merely interpersonal) dimension of affective niches based on collective intentionality (Sect. 4), we are able to combine them and address the practical question of how to distinguish exploitative from empowering niches from the perspective of participating individuals. Which forms of affective niches promote/hinder individual and collective flourishing? To make this task concrete, we focus on *social media* as a prominent platform for affective niches. First, we review social media as a type of affective niche (5.1), then we consider the empirical literature on its use and subjective well-being (SWB), and identify different causal mechanisms drawing on the social motivation hypothesis (5.2). We suggest that the findings in this literature support our claim that we-mode collectivity provides a basis on which to build more robust collective affective niches, in terms of both sources of subjective well-being and strong normative standards.

### Social media as increasingly ubiquitous affective niches

Social media, or more narrowly social networking sites (SNSs)^9^, such as Facebook, Instagram, Twitter, or WeChat and Weibo, represent an intermediate or hybrid case in relation to the two types of affective niches we discussed in Sect. 4, namely networked corporate workspace and social clubs. In that participation in social media is, in principle, voluntary in a free society, it is similar to participation in voluntary social groups. In fact, many social groups also utilize social media to facilitate both internal and external communication. At the same time, many workplaces, including universities, increasingly encourage employees to participate in and utilize social media for similar communication and dissemination purposes. In such cases participation becomes less voluntary. Many companies create an intra-network that resembles major social media platforms, and participation in such a network may well be compulsory. Moreover, behavior in SNSs may be monitored in authoritarian regimes and linked to the provision of various public and private services through the social credit system, making it practically impossible to opt out (see Creemers 2018 for a review of the social credit system in China).

Another difference between interactions in social media and those in face-to-face social groups is that the former are technologically mediated by a handful of carefully designed online platforms that dominate the market. In other words, this type of affective niche is scaffolded by both interpersonal (other participants) and material (software and hardware) elements (Krueger and Osler 2019). In terms of the three dimensions of the functionality in affective niches discussed in Sect. 2, social media, whether professional or recreational, tend to produce affective outcomes that are highly *reliable* (the participants can track others’ account identities; platforms have continuity in interfaces), *collectively structured* (platforms provide a virtual shared place for participants enabled by socio-technological scaffolding), and *highly individualized* and *entrenched* (interaction styles are customizable within a given platform’s constrained design features; users can choose specific platforms for specific purposes). As we show below, some of these functional features have interesting implications regarding the subjective well-being of those users of this particular type of affective niche.

### Subjective well-being and the use of social media

The main focus of our review in this subsection is to show (i) that social motivation is one of the basic mechanisms underlying social media use and (ii) that it can result in decreased subjective well-being. We also aim to confirm that the patterns are consistent with our claim that we-mode collectivity provides affective satisfaction more reliably than transient I-mode collectivity. For a more systematic and latest review, see Kross et al. (2021).

Why do we focus particularly on subjective well-being as a major evaluative criterion for the functioning of affective niches? Slaby, for example, discusses critical and reflective endorsement (political philosophy of mind) informed by critical cultural theory as a normative standard. Our motivation in this is our naturalistic approach to the affective niche construction, namely the position that it is significantly constrained by the configuration of basic psychological components such as social motivation, although we agree with Slaby that subjectivity is not as given contra the ‘user-resources model’. Another reason to focus on subjective well-being is to be explicit about our standards for evaluating different affective niches. Krueger and Osler (2019, Sect. 4) highlight salient features of internet-based emotional *dysregulation*, such as inter-niche conflict, manipulations by others and overreliance. Subjective well-being empirically captures these and other relevant factors for individuals (including non-affective effects). Our claim is not that subjective well-being is all that matters normatively, or that its sources are homogeneous empirically, it is rather that it captures significant effects on individuals’ affective and other satisfaction.

Subjective well-being (SWB) is operationalized and measured in different ways in psychology. These measures fall roughly into two categories, namely cognitive measure (asking judgement about one’s life satisfaction generally characterized) and affective measure (asking about one’s mood and emotions in particular temporal frames).^10^ Measured in this way, SWB is *prima facie* an indicator of a desirable state, from a subjective point of view. It also positively correlates with other major objective variables such as health, marital relationship and prosociality.

People spend a significant amount of time on social media: according to one study (Asano 2017), the average time spent per day is 76 min^11^, as opposed to 43 min of face-to-face socializing. The use of social media has become so pervasive that the research focus has shifted from *how much* (in terms of frequency and duration) people use social media to *how* (in terms of degree of engagement) (Scott et al. 2017). This was before the current pandemic made face-to-face socializing more difficult. Why do people use social media so extensively? Nadkarni and Hofmann (2012) propose what they call the dual-factor model of Facebook use: the first factor is the need to belong, or “the intrinsic drive to affiliate with others and gain social acceptance” (Baumeister and Leary 1995), and the second factor is the need for self-representation, or “impression management”. Whereas impression management more explicitly concerns the strategic aspects of situated emotions that Griffiths and Scarantino (2009) address in their model of situated affectivity, the need to belong as we interpret it is a manifestation of people’s social motivation (orientation towards affiliative stimuli in particular) as a major driver of its use. We thus focus on this factor in the following.^12^

Researchers have started studying the impact of extensive habituation in social media on SWB. The empirical picture is still incomplete, but there several interesting theories and findings are emerging from these early studies (see Kapoor et al. 2018 for a more comprehensive review of 132 studies on social media/networking, which includes a category focusing on well-being and health). The overall impact of social media use on SWB seems to depend on many things, such as: (i) the types of SWB measures (mood vs. life satisfaction; experimental vs. longitudinal; interview vs. survey vs. text-based sentiment analysis); (ii) the types of interaction on social media (post vs. chat); and (iii) the types of individuals (in terms of social comparison orientation) (Kross et al. 2021). We consider the positive and negative impacts in turn, our aim being to understand the underlying mechanisms.

#### The positive effects of social media use on subjective well-being

There is some evidence of increased SWB related to the use of social media. As Verduyn et al. (2017) report in their review article, for example, the active use of Facebook is positively correlated with SWB whereas there is a negative correlation with passive use such as browsing other people’s posts. Pittman and Reich (2016) found that image-based communication via platforms such as Instagram increased SWB relative to text-based communication via platforms such as Twitter, whereas according to Rae and Lonborg (2015), existing relationships mediate the positive effect of social media. Kim and Lee (2011) suggest that honest self-representation increases SWB via increased perceived social support of Facebook friends. It was also found in a study focused on Twitter (Bliss et al. 2012) that SWB as measured on a text-analysis-based hedonometer correlated positively among reciprocally connected individuals (i.e. who interacts in a reciprocal way), but not among those with non-reciprocal relations.

It is worth highlighting that existing reciprocal interpersonal relationships have been shown to mediate positive impacts on SWB. We interpret this as indicating that such relationships facilitate positive experience via synchrony, fellow-feeling, and emotion sharing via social media. Moreover, we believe that this pattern is consistent with our account of robust collectivity (Sect. 4.2): we suggest that using social media contributes to SWB when it forsters the creation, maintenance and enhancement of reciprocal and cooperative interpersonal relations, providing a basis for positive online experiences as phenomenologically detailed by Osler (2020).^13^

#### The negative effects of social media use on subjective well-being

There is also some evidence of decreased SWB attributable to Facebook use in particular. Verduyn et al. (2017), who report the positive impact of active use also found a more robust negative impact of passive use, which they hypothesized was attributable to social comparison and envy, whereas the positive impact reflected feelings of social connectedness. In their experimental study on the impact of browsing strangers’ (confederates’) posts on Instagram, de Vries et al. (2018) hypothesized two opposing mechanisms: emotional contagion (fellow-feeling in our terms, causing positive feelings) and social comparison (causing negative feelings). They found that individual differences in terms of a tendency to engage in social comparison predicted the reported affect after browsing positive Instagram posts. Namely, subjects with a tendency to engage in social comparison reported negative affect, whereas those with no such tendency reported positive affect. The authors also point out that photo-enhancing features of Instagram may accentuate negative effects of social comparison by biasing people towards positive self-presentation, compared to other less image-centered platforms. Sagioglou and Greitemeier (2014) propose another mechanism through which the use of social media causes negative feelings: they found a deterioration in mood after longer use of Facebook, which related to the feeling of not having done anything meaningful or having wasted their time.

Research on *moral contagion* in social media (in text-based platforms such as Twitter), although not primarily concerned with users’ subjective well-being, provides another mechanism that may give rise to the negative impact of social media use on SWB. This research is primarily interested in the psycho-technological mechanisms of *information diffusion* through which the morally and politically relevant emotional contents spread in social networks via sharing and posting (Brady et al. 2020), rather than *emotional* diffusion or contagion (see Goldenberg and Gross 2020, Box 3). However, since such contagion is mediated by the users’ appraisals of a given situation, it often affects their subjective (cognitive and affective) states. In particular, the motivation, attention, and design (MAD) model of moral contagion (Brady et al. 2020), drawing on the social-identity approach in social psychology, hypothesises that one of the key mechanisms of moral contagion is a set of motivations to maintain (i) a positive in-group image relative to out-groups and (ii) a good standing in one’s group, when one feels threats to his or her specific group identity (e.g. in partisan political contexts). The specific design of social media, such as notification to sustain attention (detailed in the A-D parts of the MAD model) could also reduce well-being. A recent study (Schöne et al. 2021) also reports that negative emotional language spreads further than positive one on social media in response to *both* negative and positive political events. The authors interpret this result as consistent with the idea that intergroup contexts make negative emotions more prevalent, and conjecture that moral contagion may negatively impact well-being, citing the evidence that daily exposure to political situations is associated with worse physical and mental well-being, and that exposure to hateful content lead to other negative consequences such as decreased social trust and exaggerated outgroup threat.

All in all, these lines of research suggesting the negative impact of social media use -- envy as a result of social comparison, a feeling of having wasted time, and exposure to negative emotional language on social media in intergroup contexts -- raise a question. Why do people *voluntarily* spend more time on social media if the resulting SWB is negative overall? In another study Sagioglou and Greitemeier (2014) found that their subjects assumed 20 min of Facebook use would make them feel better afterwards. In line with other studies, they propose an *affective forecasting error hypothesis* (cf. Wilson and Gilbert 2005). People frequently make forecasting errors, mistakenly thinking that keeping the option to change their decision at a later point, for example, would make them feel better off later, or that taking revenge would make them feel better. Similarly, Wilson and Gilbert (2005) suggest that people may erroneously predict their own feelings, in this case predicting positive emotions after Facebook use, which explains their use despite the negative experiences that tend to follow.

As in other cases of serious forecasting errors that negatively affect subjective well-being, typically in cases of substance and gambling addiction, we need to explain why the subjects themselves do not readily correct this error despite the constant feedback. According to the social motivation hypothesis, the anticipation of social reward is such a basic and strong motivator of behavior that the subjects may not be able to make a rational choice based on their all-things-considered intertemporal preferences. In other words, an occasional jackpot of positive feeling or its anticipation might make people come back to the platform, even if the overall (average) expected feeling is negative. Another possible mechanism is the motivational power of emotional sharing, or the pleasure of sentiment correspondence. This mechanism motivates affiliation on social media platforms with *relevantly similar others* in terms of hobby activities, political views and socio-economic status so as to increase affective correspondence. At the same time, however, the sorting on the platforms may make social comparison more salient and compelling, leading to what Krasnova et al. (2015) call a “self-enhancement envy spiral.” Moreover, in morally and politically relevant intergroup contexts, the MAD model suggests that such sorted in-groups tend to exaggerate outgroup threats through expressions of moral outrage motivated by the maintenance of one’s in-group reputations (Brady et al. 2000, p. 989). In both cases, social motivation drives individuals to engage in social media even though the net impact on SWB could be negative. The social-motivation hypothesis makes sense of this in construing social motivation as an evolved (i.e., fitness-enhancing) disposition of individuals as members of *homo sapiens*, whereas SWB is a major goal among *individual persons* with different biographies and culturally sanctioned aspirations in contemporary societies.

In sum, our review of the use of social media as affective niches, and of their impact on subjective well-being shows that there are multiple technology-mediated social psychological mechanisms that affect SWB both negatively and positively. We highlight the central role of social motivation in affiliation and approval, and how it interacts with other mechanisms such as social comparison and in-group reputation management. We also suggest that the robust form of collectivity characterised by we-mode collectivity (with a reciprocal and cooperative group ethos) correlates with the improved SWB that a given affective niche can afford. But beyond a narrow focus on SWB, how should we begin reflectively to evaluate the functions of social media as *collective* affective niches? We turn to this normative question in the next section.

## Normative evaluation of collective affective niche construction

Finally, we come back to Slaby’s (2016) critique and discussion on how to normatively evaluate scaffolded affective niche construction. Krueger and Osler (2019) provide a detailed account of the various ways in which the Internet-enabled technologies scaffold virtual we-spaces as interpersonal affective niches. Most importantly for our purposes and as mentioned above, they explicitly discuss how such scaffolded affective niches can cause emotional *dysregulation* due to their hyper-social and technologically mediated nature. The gist of their analysis is that as much as we trust and individualize these affective niches, crucial elements of the environment are beyond agential control, making us affectively vulnerable and open to manipulation. Commenting on social media specifically, they state:

While we might go onto Instagram to be entertained, to feel a sense of connection with our followers, those who have designed Instagram have done so in order to create a platform that makes money. They are motivated by creating a platform that is addictive and is geared toward marketing products to us. (Krueger and Osler 2019, p. 226).

Our unique contributions to this critique are twofold. First, on the theoretical level our account of social motivation in Sect. 3 and our review of the negative effects of SNSs on SWB in Sect. 4 highlight one significant way in which social media are ‘addictive’: SNS platforms are carefully engineered and designed to tap into people’s social motivation to join, spend time and come back, sometimes despite a recurring mismatch between what agents want (and anticipate having) and what they end up getting in terms of affective experiences from social media. Hence, although we should be careful not to misuse or abuse the term addiction in the context of behavioral policy (Ross et al. 2012), we believe that there is a genuine concern here on which the concept of social motivation could shed light. On the empirical level, we reiterate that the evidence on the impact of social media on SWB is mixed (both positive and negative), leading to various hypothesized mechanisms and their interactions (Sect. 5). Hence, although it is true that social media instantiate interpersonal affective niches that are partly or even largely beyond any individual user’s control, it does not follow that damage to individual well-being is the intended or expected result.

Our second contribution to the normative debate on affective niche construction is the elucidation of its distinctively collective (as opposed to merely interpersonal) dimension in terms of we-mode vs. I-mode intentionality. This distinction also serves to qualify Krueger and Osler’s (2019) implicit identification of the for-profit nature of the SNSs as the main culprit of the participants’ emotional dysregulation. We believe that the self-interested profit motive of SNSs providers as such is perfectly compatible with their providing platforms for valued collective affective niches that contribute to the well-being of users or even help them to thrive as individuals and groups. Whether or not such a system based on I-mode (or weak we-mode) collectivity functions as a community of mutual advantages (Sugden 2018) depends crucially on both the formal regulations (e.g., antitrust law, privacy law), business models (e.g., based on advertisement revenues or subscription or other service fee) and the informal norms (e.g., respect for users’ tastes, trust and trustworthiness) that govern the market structure and the participants’ behavior therein. In this sense, the problem is not profit-seeking as such, but specific market structures such as monopoly and dominance attributable to network effects (e.g., Instagram, Facebook and WhatsApp all belong to one company) and strong incentives of content providers to compete for users’ attention with affective engagement that could be detrimental to individual subjective well-being as well as to the collective ethos of civil discourse. Moreover, these specific features of collective niches and people’s behaviour therein should be evaluated and regulated based on different normative standards (e.g., from a weak sense of civility to strong altruism), depending on different degrees of collectivity of given niches: larger groups (citizens) regardless of whether one uses SNSs or which SNSs to use, groups of consumers and providers of particular services (e.g., Facebook and its users), groups of users, and sub-groups of users who share specific interests and goals (e.g., a family whose members are scattered across the world, or an activist group with a shared mission).

Finally, if an affective niche is to remain robustly collective in terms of sharing goals and values, its members need to put effort into maintaining it, independently of its merely functional performance in catering for affective needs such as a sense of connection and intimacy. In this regard, we highlight the instrumental value of *empowerment* in collectives. Although one may leave a particular voluntary niche (e.g., a Facebook group, or Facebook as a platform) if one does not endorse its ethos, in practice it is difficult to leave many functionally vital collective affective niches without paying substantial costs: examples include one’s nation state, religious group, workplace and family, and perhaps even Facebook. Members of these semi-default niches should be empowered in the sense that they play a crucial role in jointly realizing the goals, which they are able to influence (Bacharach 2006). A collective affective niche with empowered members, we suggest, also tends to satisfy their affective needs more reliably.

## Conclusion

In sum, we argue that affective niche construction plays an important role in our individual and collective flourishing. We review major developments in theories of situated and scaffolded affectivity, which build on evolutionary biology and social psychology, as well as on phenomenological traditions (Sect. 2). Our contributions to these interdisciplinary developments are two-fold. On the one hand, we explicate the *collective* dimension of interpersonal affective niches, thereby complementing the work of Colombetti and Krueger (2015). We applied the social motivation hypothesis to explain the need to construct collective affective niches in the first place, and to identify the key psychological mechanisms that underlie the process (Sect. 3). On the other hand, we discuss an explicitly normative dimension of the collective niche, drawing on Slaby’s critique of Colombetti and Krueger’s (2015) user-resource model and Tuomela’s we-mode intentionality (Sect. 4). We broaden Slaby’s advocacy of a political philosophy of mind in two ways. First, we examine a new case—the impact of social media on subjective well-being—that is more relevant to our focus on the collective dimension of affective niches than Slaby’s case of “corporate life hack” at the workplace (Sect. 5). Second, our analysis builds on the recent literature in theoretical and empirical psychology, as well as on collective intentionality, rather than the phenomenology of subjectivity. We believe that our approach complements Slaby’s work in articulating some of the ways in which a ‘life hack’ could also take place in largely voluntary affective niches such as social media. In our discussion (Sect. 6), we briefly address Krueger and Osler’s (2019) concern that the for-profit motive of SNS providers could make individuals affectively vulnerable and manipulable—one of the cases of emotional dysregulation. Instead of problematizing profit-motives of SNS providers, we propose a more general and encompassing normative recommendation based on the account of we-mode collectivity: (i) explicitly assess the shared goals and norms of a given niche, independently from their functions of satisfying individual needs to belong and stay connected; and (ii) make sure that its members are empowered to develop and realize those goals and norms together with other members.

## Data Availability

not applicable.

## Footnotes

1 This response is indirect in the sense that Krueger and Osler (2019) do not cite Slaby (2016).

2 Léon and others (2019) further distinguish between environmentally extended emotions (extending to and incorporating environmental objects and structures such as rings, notebooks and locations) and socially extended emotions (extending to and incorporating other people’s emotions). Some theorists use the Extended Mind framework to defend the ascribing of a numerically identical emotion -- a single emotion experienced by several participants -- to functionally integrated systems such as an infant-caretaker couple (e.g. Krueger 2013, 2014). Others, in turn, apply it in analysing the interpersonal entanglement of emotions and advocating qualitatively identical emotional sharing, meaning that sharing is understood as the convergence of individual emotions on the same object or event (e.g., Slaby 2014; Léon, Szanto & Zahavi 2019).

3 Note that our question is not about the ontology of the mind, or mental states (be it cognitive or affective), but about its functions and mechanisms. Colombetti and Krueger (2015, p. 1159) also note that one of the advantages of Sterelny’s (2010) framework is that it allows us to avoid rather unproductive debates on whether emotions exist inside or outside the head. See Colombetti and Roberts (2015, Sect. 3) for a more detailed discussion of Sterelny’s (2010) criticism of the extended mind thesis, and how the former can be made compatible with the latter. See also Carter et al. (2016) for a useful distinction between *the extended mind thesis* (EMT) focusing on mental states and *the hypothesis of extended cognition* (HEC) focusing on cognitive processes. We interpret that Colombetti and Krueger (2015) are motivated by HEC in drawing on Griffiths and Scarantino (2009) and Sterelny (2010). See also Stephan et al. (2014, Sect. 6).

4 Krueger and Osler (2019) discuss interpersonal affective scaffolding more thoroughly, but we leave discussion of their elaboration until later sections.

5 Our social motivation hypothesis is compatible with Gallotti et al.’s (2017) view that regards interpersonal alignment, understood in terms of dynamic and reciprocal exchange of information, rather than purposive joint action, as the paradigmatic form of social interaction. The social motivation hypothesis similarly suggests that the construction and maintenance of interpersonal and collective affective niches is intrinsically motivating social behaviour that need not aim at any joint goals.

6 We do not discuss Massively Multiplayer Online Role-Playing Games (MMORPGs) such as World of Warcraft and Everquest that may facilitate joint action for shared goals in virtual reality here (see e.g., Ekdahl and Ravn 2021).

7 We emphasize the we-mode collectivity is only a *necessary* but not yet *sufficient* condition for the normative adequacy of collective affective niches. Thus, we observe that there can be we-mode collectivity in echo chambers and epistemic bubbles with morally reprehensible values and goals in the internet. These affective niches can be criticized on moral grounds.

8 As we noted in Footnote 5 above, Bruni and Sugden (2013) argues that even a very weak I-mode collective has some kind of normative glue in the form of collectively acknowledged virtues.

9 Social media is sometimes more broadly defined as “a group of internet-based applications that build on the ideological and technological foundations of Web 2.0 and permit the continuous creation and exchange of User Generated Content (UGC)” (Kaplan and Haenlein 2010, p. 61). We will focus on the most popular social media, SNSs, and use these terms interchangeably.

10 For a more comprehensive methodological analysis of the science of well-being, see e.g. Alexandrova (2018).

11 35 min on Facebook; 25 min on Snapchat, 15 min on Instagram, 1 min on Twitter.

12 It is possible to take a less theory-driven approach, or a more emic approach as opposed to an epic approach and review reasons that social media users provide themselves. We do not draw much on this type of research, partly because of our theoretical interest in social motivation, which is nicely captured by this theory, and partly because of an empirical observation that specific reasons people provide for Facebook use do not seem to moderate the impact of Facebook use on subjective well-being (Kross et al. 2013).

13 Of course it is not ‘when and only when’. For example, Pittman and Reich’s (2016) comparison between Instagram and Twitter suggests a positive impact of non-propositional sharing, which is distinct from we-mode intentionality.
